# Comparative Evaluation of Oral Health Status in Patients Undergoing Orthodontic Treatment With Clear Aligner Therapy Versus Conventional Fixed Appliance Therapy: An Umbrella Review

**DOI:** 10.7759/cureus.103744

**Published:** 2026-02-16

**Authors:** Hemannthee Thota, Himaja Vanteddu, Hepsi Singavarapu, Hepsiba A, Pushpika Sunkara, Chaitanya Krishna Dev Pillutla

**Affiliations:** 1 Orthodontics and Dentofacial Orthopaedics, Sibar Institute of Dental Sciences, Guntur, IND; 2 College of Dentistry, Sibar Institute of Dental Sciences, Guntur, IND; 3 Orthodontics and Dentofacial Orthopaedics, Drs. Sudha and Nageswara Rao Siddhartha Institute of Dental Sciences, Vijayawada, IND; 4 Orthodontics and Dentofacial Orthopaedics, Gitam Dental College, Visakhapatnam, IND

**Keywords:** clear aligners, clear aligner therapy, conventional fixed appliance therapy, fixed orthodontic appliances, gingival health, meta-analysis, oral health, plaque-index, systematic review

## Abstract

Orthodontic treatment serves an essential function in correcting malocclusion and improving both function and esthetics, but different appliance systems can influence oral health outcomes due to variations in plaque accumulation, gingival response, and enamel demineralization. Clear aligner therapy (CAT) has gained recognition as a substitute for traditional fixed appliance therapy (CFAT), asserting advantages such as improved oral hygiene and fewer periodontal issues. This overview assesses and integrates results from current systematic reviews and meta-analyses that compare the oral health conditions of patients receiving CAT with those treated with CFAT. An extensive examination of peer-reviewed Preferred Reporting Items for Systematic Reviews and Meta-Analyses (PRISMA) guidelines-compliant meta-analyses and systematic reviews was carried out, making use of databases like PubMed, Scopus, and the Cochrane Library. Studies assessing oral hygiene parameters, including periodontal, gingival, and plaque indices, and enamel demineralization, were included. The reviews chosen were assessed based on their methodological rigor, founded on the AMSTAR-2 criteria. After eliminating 327 duplicates, there were 916 records left for title and abstract screening. Out of these, 742 records were discarded for failing to fulfill the inclusion requirements. The entire texts of 174 articles were evaluated for inclusion, with 166 being excluded because there were insufficient data, irrelevant outcomes, or poor study design. In the end, the inclusion criteria were fulfilled by eight meta-analyses and systematic reviews, which were incorporated into this overview. Across the included reviews, findings consistently showed superior oral hygiene results in individuals receiving treatment with clear aligners. Individuals using CAT exhibited significantly lower plaque and gingival indices in contrast to individuals who received fixed appliances, largely due to the removability of aligners, which enables more efficient oral hygiene practices. However, evidence regarding long-term periodontal effects and enamel demineralization remains limited and heterogeneous. Most reviews demonstrated moderate methodological quality and highlighted the necessity for uniform outcome metrics and extended follow-up durations. Typically, current research indicates that clear aligner therapy provides better short-term oral health outcomes than conventional fixed appliances. Despite these promising findings, variations in study design and a lack of long-term data underscore the necessity for further high-quality research to draw definitive conclusions about long-term oral health relating to periodontal conditions and the risk of cavities.

## Introduction and background

Orthodontic treatment plays a crucial part in correcting malocclusions, enhancing facial esthetics, and improving overall oral function. Traditionally, conventional fixed appliance therapy (CFAT) involving brackets, bands, and arch wires has been the standard approach for the movement of teeth. Nevertheless, the introduction of clear aligner therapy (CAT) has introduced a more esthetic, comfortable, and removable alternative that is increasingly preferred by both patients and clinicians [1].

Despite their shared objective of achieving optimal tooth alignment, these treatment modalities differ in their impact on the importance of oral health regarding periodontal conditions. Fixed appliances are linked to challenges in maintaining oral hygiene, leading to increased plaque accumulation, gingival inflammation, and an increased likelihood of enamel demineralization and white, spotted lesions. However, in contrast, clear aligners might offer improved oral health outcomes due to their removability, which facilitates better access for tooth brushing and flossing [2-4].

Oral health outcomes in orthodontic research are commonly evaluated using standardized indices. White spot lesions (WSLs) present clinically as opaque enamel lesions adjacent to orthodontic attachments. The plaque index (PI) assesses the quantity of dental plaque, the gingival index (GI) evaluates the severity of gingival inflammation, and bleeding on probing (BOP) serves as an objective indicator of periodontal inflammatory response.

CAT differs from fixed appliances in design and biomechanics in ways that may influence oral hygiene. The removability of aligners facilitates effective plaque control, while their smooth thermoplastic surfaces lack plaque-retentive components inherent to fixed appliances. These characteristics provide a mechanistic rationale for potential reductions in plaque accumulation, gingival inflammation, and WSL development with CAT.

The use of clear aligners has increased substantially in contemporary orthodontic practice. Randomized clinical evidence has demonstrated differences in oral-health-related quality of life between aligner and fixed-appliance therapy after one year of treatment [5]. However, evidence regarding comparative clinical effectiveness and oral-health outcomes remains inconsistent, with recent systematic reviews highlighting heterogeneity across aligner systems and malocclusion severity [6].

Understanding the different impacts of aligners on teeth is increasingly important as their popularity rises. Several studies have looked at factors like plaque and gingival indices, bleeding upon probing, periodontal health, and caries occurrence throughout orthodontic therapy with CAT compared to CFAT [7]. However, findings across the literature remain inconsistent due to variability in study design, follow-up durations, and outcome measures.

To possess the ability to tackle this uncertainty, a systematic review and meta-analysis was carried out in order to gather existing evidence and evaluate the oral and dental health circumstances of individuals currently receiving orthodontic treatment using clear aligners compared to those with fixed orthodontic appliances. This article summarizes that analysis, aiming to provide clinically relevant insights for orthodontists and dental professionals in choosing the most appropriate treatment modality concerning oral hygiene preservation.

## Review

Methodology

Study Design

This overview was performed as a systematic review and meta-analysis, adhering to the guidelines set forth by the Preferred Reporting Items for Systematic Reviews and Meta-Analyses (PRISMA). The objective aimed to assess and compare the oral health outcomes of individuals undergoing orthodontic care through CAT versus those using CFAT. There was no need for ethical approval since it did not involve individual participation, any form of intervention, or the collection of personal data. This evaluation was prospectively registered in PROSPERO under registration ID CRD420251089181. The primary outcome was the incidence or severity of WSLs, and secondary outcomes included PI, GI, and BOP during orthodontic treatment.

To ensure a comprehensive assessment of aligner therapy relative to fixed appliances, both randomized controlled trials and well-designed cohort studies were considered eligible. This approach reflects the evolving evidence base in orthodontics, where ethical, practical, and technological factors may limit the availability of long-term randomized trials. A recent systematic review evaluated Invisalign’s tooth-movement efficacy versus fixed appliances and underscores the need to consider both clinical and oral-health endpoints when comparing treatment modalities [8]. Including diverse study designs, therefore, allows for a more complete evaluation of treatment effectiveness and associated oral-health outcomes.

Eligibility Criteria

Research studies were chosen based on the PICO framework: Population (P): Individuals (both adolescents and adults) receiving orthodontic treatment; Intervention (I): Treatment utilizing clear aligners; Comparison (C): Traditional fixed appliances (metal or ceramic brackets accompanied by wires); Outcomes (O): Indicators of oral health conditions, including PI and GI, periodontal pocket depth (PPD), BOP, incidence of caries, and overall periodontal health.

Inclusion Criteria

Only systematic reviews and meta-analyses that appeared in peer-reviewed journals were deemed eligible. Research comparing CAT and CFAT was included. Research that documented at least one of the predefined oral and dental health outcomes was considered. Human studies published in English were accepted.

Exclusion Criteria

Case reports, narrative reviews, editorials, and correspondence to the editor. Studies that lack evaluation of both treatment approaches. Studies that fail to provide sufficient details for extraction or statistical analysis.

Information Sources and Search Strategy

A thorough literature review was conducted utilizing PubMed, Scopus, and the Cochrane Library to locate systematic reviews and meta-analyses assessing oral health results in patients undergoing CAT compared to those undergoing CFAT. The search encompassed publications released from January 01, 2015, to July 01, 2025.

For PubMed, the search approach integrated Medical Subject Headings (MeSH) along with free-text terms utilizing Boolean operators. The MeSH terms Orthodontic Appliances AND Oral Health were combined with free-text keywords related to orthodontic treatment modalities, including orthodontic treatment, clear aligner, aligner therapy, Invisalign, fixed orthodontic appliance, conventional braces, and brackets, using the operator OR. Results related to oral health were examined utilizing the terms oral health status, periodontal status, gingival health, plaque index, and dental caries, combined using OR. These concept groups were linked using the operator AND. The search was further restricted using the terms systematic review OR meta-analysis.

In Scopus, indexed terms and free-text keywords in titles, abstracts, and Boolean operators were used to combine the keywords. Terms related to orthodontic treatment (clear aligner OR aligner therapy OR Invisalign OR fixed appliance OR fixed braces OR brackets) were combined with oral health outcome terms (oral health OR oral health status OR periodontal health OR gingival health OR plaque OR caries) using the operator AND. The findings were restricted to systematic reviews OR meta-analyses.

In the Cochrane Library, MeSH descriptors for Orthodontic Appliances AND Oral Health were combined with free-text terms (clear aligner OR Invisalign OR fixed appliance OR fixed braces) and oral health outcome terms (periodontal status OR gingival health OR plaque OR caries) using Boolean operators. The search focused on systematic reviews OR meta-analyses.

Data Search, Retrieval, and Extraction

Two independent reviewers (H.T. and H.S.) conducted the search for relevant literature, selection of studies, retrieval of data, and extraction of data using standard forms. The reviewers individually evaluated the identified studies for eligibility while blinded to each other’s decisions. Conflicts were settled through conversation, and when a mutual agreement could not be achieved, a third reviewer (C.P) was brought in to help reach a final decision.

Data Items and Collection

The information gathered from every article included the name of the first author and the year it was published, the title of the study, and the overall count of studies included, study design, index considered, outcomes assessed, A Measurement Tool to Assess Systematic Reviews (AMSTAR)-2 score [9], and overall quality of evidence.

Quality Assessment of Individual Systematic Reviews

The standard of the methodology employed in the systematic reviews included was appraised independently by two reviewers utilizing the AMSTAR-2 Measurement Tool. Each review was rated across different AMSTAR-2 domains, and the overall confidence in their results was classified as high, moderate, low, or critically low, depending on the presence of critical and non-critical weaknesses. Any differences in the quality assessment were addressed through discussion, and if an agreement could not be reached, an additional reviewer was involved. Clinical decision‑making should integrate both efficacy and oral‑health evidence, given the variable findings across reviews and trials [5].

Risk of Bias Assessment of RCTs

The risk of bias of randomized controlled trials that were included in the review was assessed independently by two reviewers utilizing the Cochrane Risk of Bias tool. The domains reviewed included the generation of random sequences, concealment of allocation, participant and personnel blinding, blinding of the evaluation of results, incomplete data on outcomes, selective reporting of outcomes, and other possible bias sources. Every domain was categorized as having a low, high, or uncertain risk of bias. When reviewers were unable to reach a consensus, a third reviewer was consulted, and disagreements were resolved through discussion.

Summary Measures and Synthesis of Results

Summary measures and the synthesis of results were presented following PRISMA guidelines. Effect estimates, such as standardized mean differences and mean differences, and risk ratios, along with 95% confidence intervals, were gathered from the systematic reviews and meta-analyses included in the study. When a sufficient level of homogeneity was observed, pooled estimates were provided to compare oral health outcomes between clear aligner therapy and traditional fixed-appliance therapy. In circumstances where there was considerable clinical or methodological heterogeneity, a narrative synthesis was conducted. Statistical heterogeneity, when reported, was evaluated using the I² statistic, and the power of each outcome's evidence was assessed based on the standard of the included reviews.

Results 

Study Selection and Characteristics of Included Reviews

**Figure 1:**
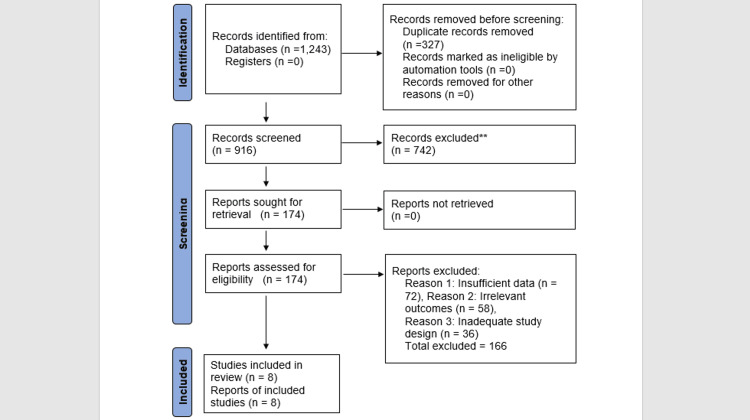
PRISMA flowchart showing the identification and selection of eligible studies at each stage PRISMA, Preferred Reporting Items for Systematic Reviews and Meta-Analyses

A sum of 1,243 records were found in PubMed (n=462), Scopus (n=538), and the Cochrane Library (n=243). After discarding 327 duplicates, 916 records moved on to title and abstract screening. Of these, 742 were excluded because they didn't meet eligibility criteria. Full-text evaluation was conducted on 174 articles, and 166 were removed due to a lack of sufficient data, irrelevant outcomes, or inadequate study design. In the end, eight systematic reviews and meta-analyses were included in this overview. These eight reviews were released in the years 2018 through 2025 and collectively examined oral health results for individuals receiving CAT in contrast with CFAT. The reviews included a combination of randomized controlled trials (RCTs), cohort studies, cross-sectional designs, and clinical trials. The sample sizes and follow-up durations varied significantly among the reviews, leading to methodological heterogeneity (Figure 1).

Characteristics of Included Reviews

The eight reviews examined were published from 2018 to 2025 and collectively assessed oral health outcomes in individuals undergoing CAT in comparison to CFAT. These reviews included a variety of study designs, such as RCTs, cohort studies, cross-sectional studies, and clinical trials. There was significant variation in sample sizes and follow-up periods across the reviews, leading to differences in methodology.

Lu et al. and Jiang et al. primarily examined the plaque and gingival index, periodontal measures, while Oikonomou et al. and Raghavan et al. focused on enamel demineralization, plaque buildup, and caries-related bacteria. Llera-Romero et al. included broader factors, such as microbial changes and the existence of white spot lesions. Di Spirito et al. conducted an umbrella review that aggregated findings from various systematic reviews. Crego-Ruiz and Jorba-García assessed periodontal health and gingival recession, and Dipalma et al. compared overall periodontal outcomes between aligners and fixed orthodontic appliances [10-17].

In summary, all eight reviews tackled oral hygiene elements, periodontal health, or enamel integrity, offering a thorough basis for assessing the comparative effects of CAT and CFAT (Table 1).

**Table 1:** Analysis of studies included in this overview

S. No	Author and Year	Title	Article Type	No. of Studies	Study Design	Index Considered	Outcome Assessed
1.	Haili Lu (2018) [10]	Assessment of the periodontal health status in patients undergoing orthodontic treatment with fixed appliances and the Invisalign system: a meta-analysis	Systematic Review and Meta-Analysis	7	Prospective cohort study (7)	Gingival index (GI), PLI, sulcus probing depth (SPD), and sulcus bleeding index (SBI)	Comparing with the traditional fixed appliances, patients treated with Invisalign have better periodontal health
2.	Qian Jiang (2018) [11]	Periodontal health during orthodontic treatment with clear aligners and fixed appliances: a meta-analysis	Systematic Review and Meta-Analysis	9	Cohort study (6), Randomized controlled trial (3)	Plaque index (PI), gingival index (GI), probing depth (PD), for periodontal bleeding evaluation, including papillary bleeding index (PBI), sulcus bleeding index (SBI), bleeding on probing (BOP), and nonbleeding papillae after probing.	Clear aligners were better for periodontal health than fixed appliances and might be recommended for patients at high risk of developing gingivitis.
3.	Elissaios Oikonomou (2021) [12]	Impact of aligners and fixed appliances on oral health during orthodontic treatment: a systematic review and meta-analysis	Systematic Review and Meta-Analysis	21	Prospective clinical trial (11), randomised controlled trial (6), cross-sectional (1), retrospective cohort (3)	Gingival index (GI), plaque index (PI), bleeding on probing (BoP), probing depth (PD), clinical attachment loss (CAL), recession, the concentration of cariogenic and periodontal microflora in the surrounding tissues, as well as formation of incipient (i.e., white lesions) or advanced caries lesions.	Patients treated with aligners and no additional attachments/adjuncts presented potentially higher levels of oral health overall
4.	Shailaja Raghavan (2023) [13]	White spot lesions, plaque accumulation, and salivary caries-associated bacteria in clear aligners compared to fixed orthodontic treatment. A systematic review and meta-analysis	Systematic Review and Meta-Analysis	14	RCT (5), prospective cohort (7), concomitant trial (1), retrospective Cohort (1)	Mean plaque index score (modified index of Loe and Silness, Turesky Modified Quigley Hein Plaque Index, Modified plaque index of Loe) Digital photographs, Quantitative Light Fluorescence, visual examination, quality of life (QoL)	Less plaque accumulation and less salivary caries-associated bacteria in clear aligners, which might be related to the reduced incidence and severity of WSLs associated with clear aligners when compared with conventional fixed appliances
5	Ana Sandra Llera-Romero (2023) [14]	Periodontal health status, oral microbiome, whitespot lesions, and oral health related to quality of life: clear aligners versus fixed appliances: a systematic review, meta-analysis, and meta-regression	Systematic Review, Meta-Analysis, and Meta-Regression	31	Randomized clinical trial (6), retrospective study (4), prospective/cohort study (14), cross-sectional study (4), non-randomized clinical trial (3)	Sulcus bleeding index; Gingival index; papilla bleeding index; visual analogic scale; pocket probing depth; gingival recessions; Gingival bleeding index; Basic periodontal exam index; simplified plaque index; simplified gingival index.	Patients wearing clear aligners show better periodontal indicators, less risk of white spot development, less biofilm mass, and a better quality of life than patients with conventional fixed orthodontics.
6	Federica Di Spirito (2023) [15]	Impact of clear aligners versus fixed appliances on periodontal status of patients undergoing orthodontic treatment: a systematic review of systematic reviews	Systematic Review of Systematic Reviews	4	Systematic review (4)	Plaque index (PI), gingival index (GI), periodontal probing depth (PPD), and sulcus bleeding index (SBI)	The impact of orthodontic treatment with clear aligners and fixed appliances on periodontal health should be considered comparable, and there is no evidence to support the choice of clear aligners as the first treatment option in patients at risk for gingivitis or periodontitis.
7	Marina Crego-Ruiz (2023) [16]	Assessment of the periodontal health status and gingival recession during orthodontic treatment with clear aligners and fixed appliances: a systematic review and meta-analysis	Systematic Review and Meta-Analysis	12	Randomized clinical trials (3), prospective cohort studies (8), retrospective cohort study (1)	Plaque index (PI), gingival index (GI), periodontal probing depth (PPD), bleeding on probing (BoP)	To date, there is not enough evidence to conclude that CA maintains better periodontal health during orthodontic treatment than FA.
8	Gianna Dipalma (2025) [17]	The differential impact of clear aligners and fixed orthodontic appliances on periodontal health: a systematic review.	Systematic Review	8	Randomized trial (1), cross-sectional study (2), comparative cross-sectional (1), observational study (1), comparative study (1), prospective clinical study (1), prospective pilot study (1)	Gingival index (GI), sulcus bleeding index (SBI), plaque index (PI), gingival recession (REC), bleeding on probing (BOP), probing depth (PD)	Clear aligners offer advantages over fixed appliances in terms of enhancing periodontal health, improving patient compliance, and providing long-term benefits, particularly in patients with severe periodontitis. The effectiveness of clear aligners is linked to better management of periodontal complications and overall oral hygiene.

Oral Hygiene and Plaque Accumulation

All the reviews mentioned consistently indicated that patients under clear aligner therapy experienced less plaque buildup. Lu et al. and Jiang et al. observed substantially reduced plaque index scores among users of clear aligners, primarily because of the fact that the aligners can be removed, allowing for uninterrupted brushing and flossing. Oikonomou et al. supported these conclusions through a pooled analysis, showing statistically better plaque management with clear aligners [10-12].

Raghavan et al. found a decrease in the accumulation of cariogenic bacteria in patients with aligners, further reinforcing the notion of improved oral hygiene. Similarly, Dipalma et al. and Llera-Romero et al. noted positive hygiene outcomes for users of clear aligners, highlighting enhanced accessibility for effective plaque removal. Altogether, the findings indicate a consistent benefit of clear aligners in achieving lower plaque levels while undergoing orthodontic treatment [13,14,17].

Gingival Health

Five studies indicated that people who received treatment with aligners exhibited significantly reduced gingival inflammation. Both Lu et al. and Jiang et al. observed lower gingival index scores and less bleeding upon probing in users of clear aligner therapy (CAT). Oikonomou et al. reported similar findings, demonstrating enhanced gingival conditions throughout the treatment process [10-12].

Crego-Ruiz and Jorba-García noted a decrease in gingival recession and improved gingival health indicators when comparing CAT to CFAT. Additionally, Llera-Romero et al. showed that aligner patients experienced diminished gingival inflammation. Although there was variability in the measurement tools utilized, the overall trend in the reviews supported CAT for better short-term gingival health [14,16].

Periodontal Status

Periodic outcomes, such as probing depths, attachment levels, and overall periodontal stability, were examined across multiple reviews, yielding varied results. Jiang et al. and Lu et al. reported slightly improved periodontal parameters among CAT users, although these differences did not consistently achieve statistical significance [10,11].

Crego-Ruiz and Jorba-García identified a lower risk of gingival recession in patients using aligners, indicating a more favorable response from the periodontal standpoint. Di Spirito et al. pointed out that while aligners seem advantageous for preserving periodontal stability, evidence for long-term effects is still limited. Oikonomou et al. stressed the inconsistency in periodontal evaluations, which hinders the ability to establish strong long-term conclusions. In summary, short-term periodontal outcomes tend to favor CAT, but the long-term implications are still uncertain [12,15,16].

Enamel Demineralization and Caries Development

Multiple reviews highlighted the distinctions in enamel demineralization between CAT and CFAT. Raghavan et al. found that CAT users had a notably lower WSL incidence, which correlated with less accumulation of plaque around brackets and wires. Similarly, Llera-Romero et al. and Oikonomou et al. reported lower rates of enamel demineralization among patients using aligners [12-14].

While the reviews consistently indicated fewer WSLs in CAT users, Dipalma et al. and Jiang et al. mentioned that the evidence was heterogeneous due to differing diagnostic criteria and timing of assessments. Nevertheless, the general trend suggests that CAT may lower the risk of caries and enamel demineralization in comparison to CFAT [11,17].

Methodological Quality

The methodological quality across the reviews varied. Most were rated as moderate quality according to AMSTAR-2 criteria, with some elements being of low quality due to incomplete reporting on risk-of-bias assessments, protocol registrations, or management of heterogeneity.

Di Spirito et al. conducted an umbrella review that provided the most detailed methodological evaluation but also pointed out inconsistencies among the primary studies. Oikonomou et al. and Llera-Romero et al. showed stronger methodological rigor, utilizing structured search strategies and thorough bias evaluations. In contrast, earlier reviews like those by Lu et al. and Jiang et al. had limitations characteristic of the earlier literature on CAT, such as smaller sample sizes and insufficient long-term data [10-11,14,15].

Quality of Evidence: Risk of Bias

The studies reviewed included systematic reviews and meta-analyses published between 2018 and 2025, which included randomized controlled trials and observational studies. The main tools employed for assessment of periodontal outcomes were plaque and gingival indices, bleeding on probing, depth of probing, and sulcus bleeding index. The overall quality of the evidence was moderate.

There was methodological heterogeneity among the studies regarding design, sample size, follow-up duration, and outcome reporting. Most reviews indicated a trend towards better gingival health and control of plaque with the clear aligners, although some reported similar outcomes between aligners and fixed appliances. The variations in study designs and outcome measures affected the consistency of findings. The risk of bias varied, with several reviews including non-randomized studies and short follow-up periods, which potentially impacted effect estimates (Table 2).

Discussion

Although aligner therapy is often associated with improved oral hygiene access compared with fixed appliances, substantial heterogeneity exists across studies evaluating aligner effectiveness and oral-health outcomes. Differences in aligner auxiliaries and attachments can modify force systems and oral hygiene access, contributing to heterogeneity across studies [18]. Variations in attachment design, auxiliary use, and treatment protocols may influence plaque retention, gingival response, and the predictability of tooth movement, complicating direct comparisons between aligners and fixed appliances.

The purpose of this overview was to consolidate the outcomes of systematic reviews and meta-analyses that examined oral health outcomes in individuals undergoing CAT as opposed to CFAT. A recurring trend observed across the reviews indicates that clear aligners tend to result in better short-term oral and periodontal health. This finding is consistent with the conclusions drawn by Lu et al., Jiang et al., and Oikonomou et al., who collectively observed significantly lower levels of plaque accumulation and gingival inflammation among users of CAT [10-12]. The ability to remove aligners enables patients to uphold improved dental hygiene, a factor that seems to significantly contribute to the reported benefits in periodontal health.

Results from other included reviews further corroborate this advantage. Raghavan et al. showed diminished levels of cariogenic bacteria and a decrease in WSLs among CAT users, bolstering the hypothesis that improved access to oral hygiene lowers the chances of enamel demineralization. Likewise, Llera-Romero et al. found better periodontal metrics and less disturbance to the oral microbiome in individuals undergoing treatment with aligners. Di Spirito et al., in their review of systematic reviews, reiterated these findings and highlighted that the overall evidence points in favor of aligners concerning periodontal health, although with significant methodological discrepancies across studies [13-15].

Further recent reviews stress the periodontal benefits of CAT. Crego-Ruiz and Jorba-García observed reduced gingival recession and enhanced periodontal indices in users of aligners, while Dipalma et al. reported consistent decreases in plaque and gingival indices, and bleeding on probing in comparison to fixed appliances. Collectively, these eight systematic reviews provide a solid basis supporting the favorable short-term oral health consequences linked to CAT [16,17].

Beyond these core findings, numerous other studies have also contributed to the growing understanding of oral health differences between CAT and CFAT. These studies collectively confirm better plaque control [18-22], reduced gingival inflammation [23-25], lower incidence of white-spot lesions [26-29], reduced periodontal pocket depth progression [24,25], improved patient-reported oral hygiene behaviors [30-32], and more favorable microbial profiles associated with aligners [33-35]. While not all of these research studies were systematic reviews, their consistent results strengthen the biological plausibility and clinical relevance of the patterns observed in the eight included reviews.

Despite this overall trend, several limitations should be acknowledged. First, as highlighted by multiple authors, including Lu et al., Oikonomou et al., and Di Spirito et al., heterogeneity in study designs, outcome measures, and follow-up durations poses challenges in directly comparing findings across investigations. Many reviews focused on short-term outcomes, with limited evidence on long-term periodontal changes or caries progression. The lack of standardized definitions for periodontal parameters, such as probing depth thresholds and plaque scoring systems, further complicates pooled analyses and may contribute to inconsistent conclusions across reviews [10,12,15].

Another limitation relates to patient selection and treatment characteristics. CAT users are often older, more motivated, or better at maintaining dental hygiene instructions, factors that may independently influence outcomes. Several reviews acknowledged possible compliance-related confounding but could not accurately account for it due to inconsistent reporting in primary studies [11,14,15]. Additionally, differences in aligner systems, treatment duration, and use of attachments or interproximal reduction may also influence periodontal or enamel responses.

Emerging evidence suggests that modified aligner systems may further influence both clinical outcomes and patient experience. Modified aligner designs with integrated springs may alter plaque accumulation and patient experience compared with standard aligners or fixed appliances [36]. These findings highlight that aligner therapy should not be considered a homogeneous intervention, and differences in appliance design may have important implications for oral-health-related quality of life and periodontal outcomes.

Nevertheless, this overview underscores an important practical implication: the removable nature of aligners consistently allows for superior hygiene maintenance compared with fixed appliances. This advantage is especially important clinically for patients who have pre-existing periodontal concerns or poor oral hygiene habits, where aligners may offer a safer therapeutic alternative. However, caution is warranted before generalizing these conclusions to long-term periodontal stability or caries prevention, as the current evidence base is insufficiently mature to demonstrate sustained benefits.

Future research should prioritize long-term, well-designed randomized controlled trials and standardized periodontal and enamel demineralization assessments. Greater uniformity in reporting outcomes, as recommended by several of the included reviews, would facilitate more accurate comparisons and stronger meta-analytic conclusions [12,14,15]. Additionally, investigations exploring microbiome changes, patient-reported oral health behaviors, and the impact of emerging aligner technologies would further enrich the field.

In summary, the synthesized evidence indicates that aligner therapy offers superior short-term oral health benefits compared with traditional fixed appliances, particularly concerning plaque control, gingival health, and the risk of demineralization of enamel. Despite their potential, these results should be interpreted cautiously due to methodological limitations and limited long-term data. Continued high-quality research is essential to determine whether these advantages persist throughout and after orthodontic treatment.

## Conclusions

Recent studies indicate that clear aligner therapy provides better short-term oral health advantages compared to traditional fixed appliance therapy. Individuals receiving therapy with aligners typically display lower levels of plaque, less inflammation of the gingiva, and a lower likelihood of enamel demineralization. These results emphasize the capability of aligners to reduce common oral health concerns related to orthodontic treatment. Nonetheless, the limitations of the present study comprise brief follow-up durations, varied methodologies, and inconsistent reporting, highlighting the necessity for well-structured, long-term randomized controlled studies. Future investigations should implement standardized outcome metrics and assess periodontal health, caries risk, and microbial changes over prolonged treatment intervals. Despite these constraints, the current data indicate that clear aligner therapy could be a beneficial choice for patients who prioritize maintaining oral hygiene while undergoing orthodontic therapy.
